# Beyond *BRAF^V600E^*: Investigating the Clinical and Genetic Spectrum of Langerhans Cell Histiocytosis in Children

**DOI:** 10.1002/cam4.70532

**Published:** 2024-12-23

**Authors:** Xue Tang, Ju Gao, Xia Guo, Zhi Wan, Jing‐jing Sun

**Affiliations:** ^1^ Department of Pediatrics, West China Second University Hospital Sichuan University Chengdu China; ^2^ Key Laboratory of Birth Defects and Related Diseases of Women and Children, Ministry of Education Sichuan University Chengdu China

**Keywords:** *BRAF*, children, clinical features, Langerhans cell histiocytosis, *MA2PK1*

## Abstract

**Background:**

Langerhans cell histiocytosis (LCH) is the most prevalent histiocytic disorder in pediatric populations, with a highly heterogeneous clinical presentation. Currently, the correlation between clinical phenotypes and molecular alterations in childhood LCH, besides the *BRAF* ^*V600E*^ mutation, has not been sufficiently studied.

**Methods:**

This study presented data on 33 pediatric LCH patients treated at our center who exhibited various molecular alterations other than the *BRAF* ^*V600E*^ mutation. Additionally, we comprehensively reviewed pediatric LCH cases with non‐*BRAF* ^*V600E*^ molecular alterations reported from January 2010 to August 2024.

**Results:**

A total of 309 pediatric LCH patients with molecular alterations beyond *BRAF* ^*V600E*^ were enrolled in the study, among whom 33 were from our center. In these LCH cases, 49 kinds of *MAP2K1* mutations, 31 kinds of *BRAF* mutations, and 4 kinds of *ARAF* mutations were found. At our center, two patients with multisystem LCH with risk organ involvement, both with *BRAF* ^*N486_P490del*^ mutation, showed poor response to induction chemotherapy for 6 weeks. Among the 303 LCH patients with *MAP2K1* or other *BRAF* alterations, patients with the *MAP2K1* mutation had a higher prevalence of single‐system bone involvement (SS‐bone) than patients carrying the *BRAF* mutation (*p* = 0.0072). Within the *MAP2K1* group, exon 3 mutations exhibited a stronger association with SS‐bone than exon 2 mutations (*p* = 0.042). Additionally, patients with the *BRAF* exon 15 mutation and *MAP2K1* exon 2 mutation had higher rates of LCH onset before age 3 compared with patients carrying the *BRAF* exon 12 mutation and *MAP2K1* exon 3 mutation (*p* = 0.037; *p* = 0.0015). Patients carrying the *BRAF* mutation in exon 15 had higher rates of liver involvement compared with patients carrying the exon 12 (*p* = 0.042).

**Conclusions:**

Pediatric LCH patients often carry recurrent somatic *MAP2K1* and *BRAF* mutations, which are associated with clinical manifestations.

**Abbreviations**
AD: active disease
LCH: langerhans cell histiocytosis
MAPK: mitogen‐activated protein kinase
MS‐LCH: multisystem LCH
NAD: non‐active disease
OS: overall survival
PFS: progression‐free survival
RO: risk organs
SS‐bone: single‐system bone involvement
SS‐LCH: single‐system LCH
SS‐MFB: multifocal bone disease
SS‐UFB: unifocal bone disease

## 1 Background

Langerhans cell histiocytosis (LCH) represents the most prevalent histiocytic disorder in pediatric populations, with incidence rates ranging from 3 to 9 cases per million individuals [1]. Currently, LCH is categorized as an inflammatory myeloid neoplasm, characterized by the clonal expansion of myeloid precursors that differentiate into CD1a^+^/CD207^+^ cells within lesions, resulting in multiple organ involvement and dysfunction [2, 3]. In pediatric patients, the clinical features of LCH are highly heterogeneous, attributed to the accumulation of mononuclear phagocytes across various tissues and organs [4]. In terms of clinical classification, LCH is categorized based on the degree of organ and system involvement. This system categorizes cases into single‐system LCH (SS‐LCH), where one organ or system is involved (further divided into uni‐focal or multi‐focal SS‐LCH), and multisystem LCH (MS‐LCH), where two or more organs or systems are involved. These categories are further stratified based on the involvement of risk organs (RO) into high‐risk (RO+) and low‐risk (RO‐) groups [5]. However, the underlying biological mechanisms for the clinical heterogeneity in childhood LCH are poorly understood.

In 2010, Badalian‐Very et al. [6] reported that 57% of LCH patients carry the oncogenic *BRAF* ^*V600E*^ mutation. Since then, several studies have demonstrated that LCH is associated with multiple gene mutations, especially those linked to the mitogen‐activated protein kinase (MAPK) signaling pathway. Notably, up to 50% of LCH patients carry the *BRAF* ^*V600E*^ mutation, alongside other activating mutations in the *BRAF*, *MAP2K1*, *ARAF*, *ERBB3*, *NRAS*, and *KRAS* genes [4, 7]. In pediatric LCH, the presence of the *BRAF* ^*V600E*^ mutation has been correlated with high disease severity and incidence of resistance to first‐line therapy [8]. Furthermore, pediatric LCH patients harboring the *BRAF* ^*V600E*^ mutation are susceptible to clinical neurodegenerative LCH [9]. However, unlike the *BRAF* ^*V600E*^ mutation, the clinical characteristics of recurrent mutations in *MAP2K1* or other *BRAF* loci remain largely unknown. Therefore, it is imperative to elucidate the genotype–phenotype correlations in childhood LCH beyond *BRAF* ^*V600E*^. For rare diseases such as LCH, it is necessary to integrate research data on genotype–phenotype correlations. In this study, we present a cohort of 33 pediatric patients diagnosed with LCH exhibiting molecular alterations beyond the *BRAF* ^*V600E*^ mutation. A comprehensive review of previously reported cases of childhood LCH involving *MAP2K1* or other *BRAF* loci is also performed. This study aimed to enhance the current understanding of the pathogenesis of this clinically heterogeneous disorder.

## 2 Methods

This retrospective study enrolled pediatric patients diagnosed with LCH from January 2013 to August 2024 at the West China Second University Hospital of Sichuan University. The diagnosis of LCH was confirmed through biopsy, based on the pathological characteristics, and was further validated by positive CD1a and/or langerin staining in accordance with the Histiocyte Society criteria [10]. Patients lacking the *BRAF* ^*V600E*^ mutation were subjected to a 47‐gene panel analysis (Table S1) using genomic DNA extracted from formalin‐fixed, paraffin‐embedded (FFPE) tissues. Finally, a cohort of 33 patients with alterations in *MAP2K1*, *BRAF*, *ARAF*, *KRAS*, and *MAP3K1* genes were enrolled in this study. Written informed consent was obtained from guardians of the patients. The study was approved by the Ethics Committee of the West China Second University Hospital of Sichuan University (Approval No.164).

At our center, first‐line therapy was administered based on the Modified‐LCH‐III chemotherapy protocol, while second‐line therapies included cytarabine, cladribine, or targeted drugs [11]. The clinical classification system for LCH categorized the disease into SS‐LCH, RO‐ MS‐LCH, and RO+ MS‐LCH. Liver, spleen, and hematopoietic system were classified as ROs [5]. The initial treatment response was evaluated at the end of 6 weeks and/or 12 weeks of induction therapy. The response to treatment was defined according to the guidelines established by the International LCH Study Group [5], which was classified into non‐active disease (NAD), active disease (AD)/better, AD/intermediate, and AD/worse. In the analysis, AD/intermediate and AD/worse were considered poor responses. The final analysis was performed on August 1, 2024, with a median follow‐up duration of 14 months (range: 4–75 months). Clinical data were collected from 33 patients, comprising variables such as age, sex, affected organs, genetic analysis, initial treatment response, and survival status.

Furthermore, all accessible English studies on non‐*BRAF* ^*V600E*^ mutations in pediatric LCH patients were comprehensively searched in various databases, including the Web of Science and PubMed. The following search terms were used for the literature search: “Langerhans cell histiocytosis,” “*MAP2K1*,” “*BRAF*,” “*ARAF*,” “genomic,” “genetic,” “mutant,” “mutation,” “alterations,” “molecular,” or “targeted therapy,” from January 2010 to August 2024. Additional studies were identified by manually searching the reference lists of the retrieved studies. Duplicate publications were excluded. Publications were deemed eligible if they provided adequate data on *MAP2K1*, *BRAF*, and *ARAF* mutations in LCH patients. Patients were included if they exhibited molecular alterations and provided the corresponding clinical information. Relevant data were extracted from the full texts and analyzed using descriptive statistics. In total, 23 publications [1, 4, 12, 13, 14, 15, 16, 17, 18, 19, 20, 21, 22, 23, 24, 25, 26, 27, 28, 29, 30, 31, 32] were included, which comprised 276 cases [4, 24, 25, 26, 27, 28, 29, 30, 31, 32] with non‐*BRAF* ^*V600E*^ molecular alterations. The association of these molecular changes with clinical phenotypes was explored.

The retrieved data were analyzed utilizing SPSS version 22.0 (IBM SPSS software, USA) and GraphPad Prism version 8 (GraphPad Software, San Diego, USA). For categorical variables, the chi‐square test, continuity correction chi‐square test, and Fisher's exact test were employed. Survival analysis was performed using Kaplan–Meier curves. Statistical significance was defined by a *p*‐value of less than 0.05. Statistically significant differences are indicated by asterisks (*p < 0.05, **p < 0.01).

## 3 Results

### 3.1 Molecular and Clinical Characteristics and Outcomes of 33 Patients From Our Hospital

**FIGURE 1:**
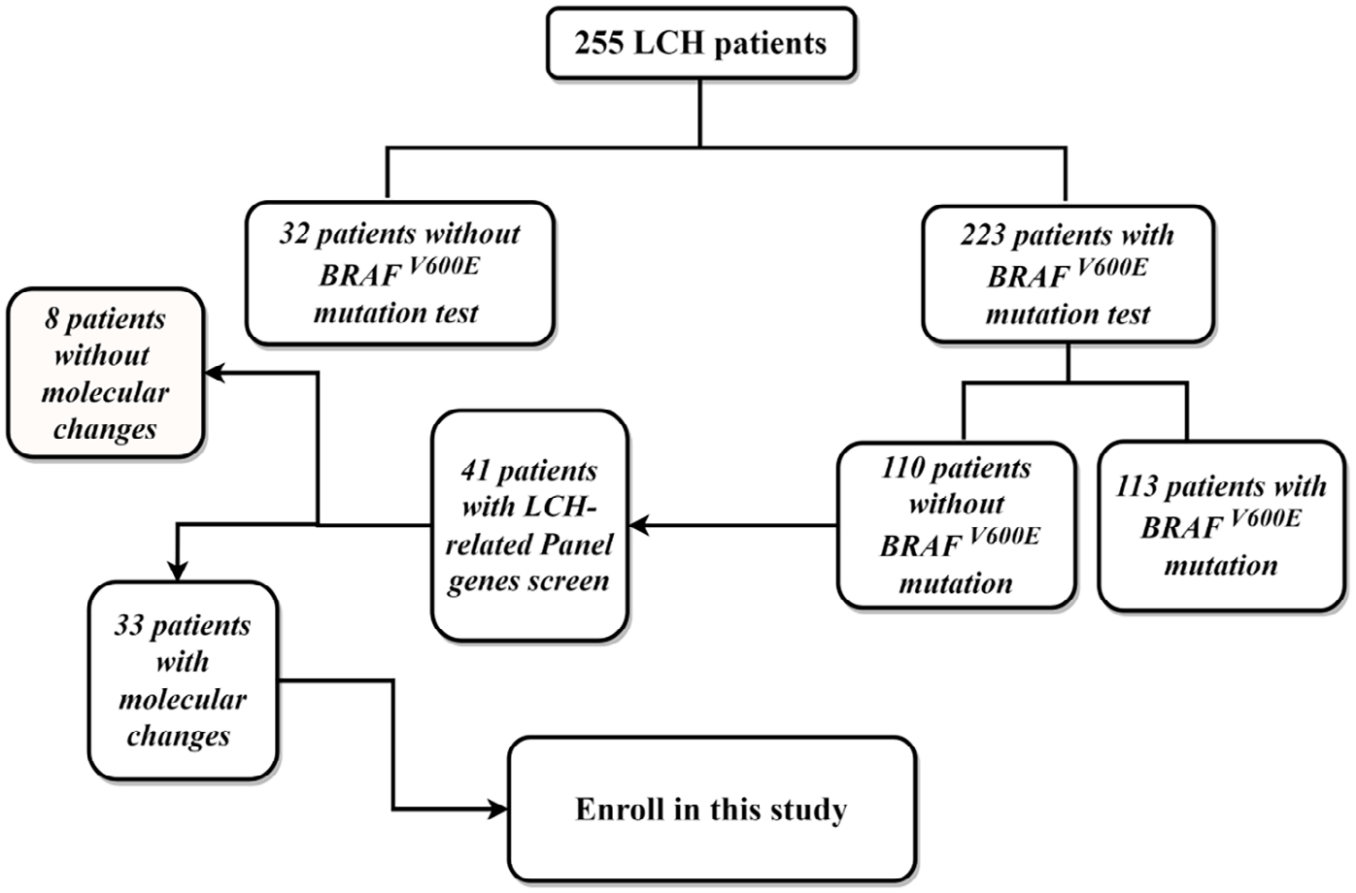
A flowchart of LCH patient recruitment in the study.

A total of 255 pediatric cases of LCH were diagnosed by biopsy and admitted to the West China Second University Hospital of Sichuan University between January 2013 and August 2024. Among them, 223 were tested for the *BRAF* ^*V600E*^ mutation using biopsy tissue, with 113 patients harboring the mutation. Among the 110 patients without the *BRAF* ^*V600E*^ mutation, biopsy samples from 41 individuals were analyzed for alterations in 47 LCH‐related genes. The findings revealed that 33 of these patients had molecular changes beyond the *BRAF* ^*V600E*^ mutation, which triggered the activation of the MAPK signaling pathway (Figure 1).

Aberrant genes identified in the 33 patients are listed in Table 1 and Table S2. Specifically, 19 patients carried *MAP2K1* mutations, 12 patients had other‐*BRAF* mutations, 1 patient had *MAP3K1* and *BRAF* mutations, 1 patient had a *KRAS* mutation, and 1 patient had an *ARAF* mutation. Moreover, 14 exhibited deletion mutations, 13 had deletion–insertion mutations, 6 had missense point mutations, and 1 had an insertion mutation. In the cohort, the *BRAF* mutation predominantly occurred at exon 12, detected in 10 patients. Conversely, the *MAP2K1* mutation was most frequent in exon 2, affecting 16 patients, whereas mutations in exon 3 were detected in 3 patients.

**TABLE 1:** The mutation sites and clinical classification groups of LCH patients with non‐*BRAF* ^*V600E*^ mutations at our center.

Gene	Exon location	Nucleotide position	Amino acid position	Numbers	Clinical classification group
*BRAF*	12	c.1455_1469del	p.L485_490delinsF	4	50% SS‐LCH 50% RO− MS‐LCH
	12	c.1457_1471del	p.N486_P490del	2	100% RO+ MS‐LCH
	12	c.1458_1472del	p.N486_T491delinsK	3	100% SS‐LCH
	12	c.1511_1517+2dup	p.R506_K507insLLR	1	RO− MS‐LCH
	13	c.1690A>G	p.M564V	1	SS‐LCH
	15	c.1799_1800delinsAT	p.V600D	1	RO+ MS‐LCH
*MAP2K1*	2	c.167A>C	p.Q56P	2	50% SS‐LCH 50% RO− MS‐LCH
	2	c.159_173del	p.F53_Q58delinsL	4	25% SS‐LCH 25% RO− MS‐LCH 50% RO+ MS‐LCH
	2	c.171_185del/c.173_187del	p.Q58_E62del	9	77.8% SS‐LCH 22.2% RO− MS‐LCH
	2	c.165_179del	p.Q56_V60del	1	SS‐LCH
	3	c.303_308del/c.304_308del	p.E102_I103del	2	100% SS‐LCH
	3	c.305_311delinsG	p.E102_K104delinsG	1	SS‐LCH
*MAP3K1*	1	c.2012C>T	p.A671V	1	RO− MS‐LCH
*KRAS*	2	c.35G>C:	p.G12A	1	RO− MS‐LCH
*ARAF*	10	c.1046_1051del	p.Q349_F351delinsL	1	RO− MS‐LCH

*Note:* One case carried mutations in the *BRAF* and *MAP3K1* genes.

The enrolled cohort comprised 23 males and 10 females. Among them, 42.4% (14 out of 33) were under 3 years old, whereas 57.6% (19 out of 33) were over 3 years old. Lesions were detected in various locations: bone lesions in 28 patients, lymph node lesions in 7 patients, lung lesions in 7 patients, skin lesions in 4 patients, liver lesions in 4 patients, thymus lesions in 2 patients, spleen lesions in 2 patients, and hematopoietic system lesions in 2 patients. Notably, the two patients who showed hematopoietic system involvement shared the same deletion–insertion mutation in *MAP2K1*, specifically F53_Q58delinsL. At the time of diagnosis, 18 patients (54.5%) had a diagnosis of SS‐LCH, while 15 patients (45.5%) had a diagnosis of MS‐LCH, with 5 patients exhibiting RO involvement and 10 patients without RO involvement (Table 1). Three patients with the *BRAF* ^*N486_T491delinsK*^ mutation were classified as SS‐LCH, while two patients with the *BRAF* ^*N486_P490del*^ mutation were classified as RO+ MS‐LCH. For patients with *the MAP2K1* ^*Q58_E62del*^ mutation, 77.8% (7 out of 9) were classified as SS‐LCH, while 75% (3 out of 4) of patients with the *MAP2K1* ^*F53_Q58delinsL*^ mutation were classified as MS‐LCH.

In the cohort of 33 patients diagnosed with LCH, 30 (90.9%) received chemotherapy, while the other three were managed with observation alone. At the last follow‐up, 31 of the 33 children were alive, including three children who had not undergone chemotherapy, 15 who completed their treatment, and 11 who were still undergoing maintenance chemotherapy. Four cases of disease relapse were recorded, two of which succumbed: one due to fulminant myocarditis while undergoing chemotherapy and the other due to tumor progression resulting in liver failure. Interestingly, poor response was observed in only 2 patients (7.7%) with *BRAF* ^*N486_P490del*^ following 6 weeks of induction chemotherapy. One patient succumbed to tumor progression resulting in liver failure despite treatment with cytarabine, cladribine, or dabrafenib. The other patient enrolled in a clinical trial investigating the efficacy of FCN‐159 (a novel MEK inhibitor) and achieved sustained no evidence of disease (NAD) due to reactivation of disease after induction chemotherapy. The estimated 2‐year overall survival (OS) and progression‐free survival (PFS) for the 33 patients were 90.9% (95% CI, 83.1–98.7) and 83.5% (95% CI, 71.4–95.7), respectively (Figure S1).

### 3.2 A Review of Scientific Literature

**FIGURE 2:**
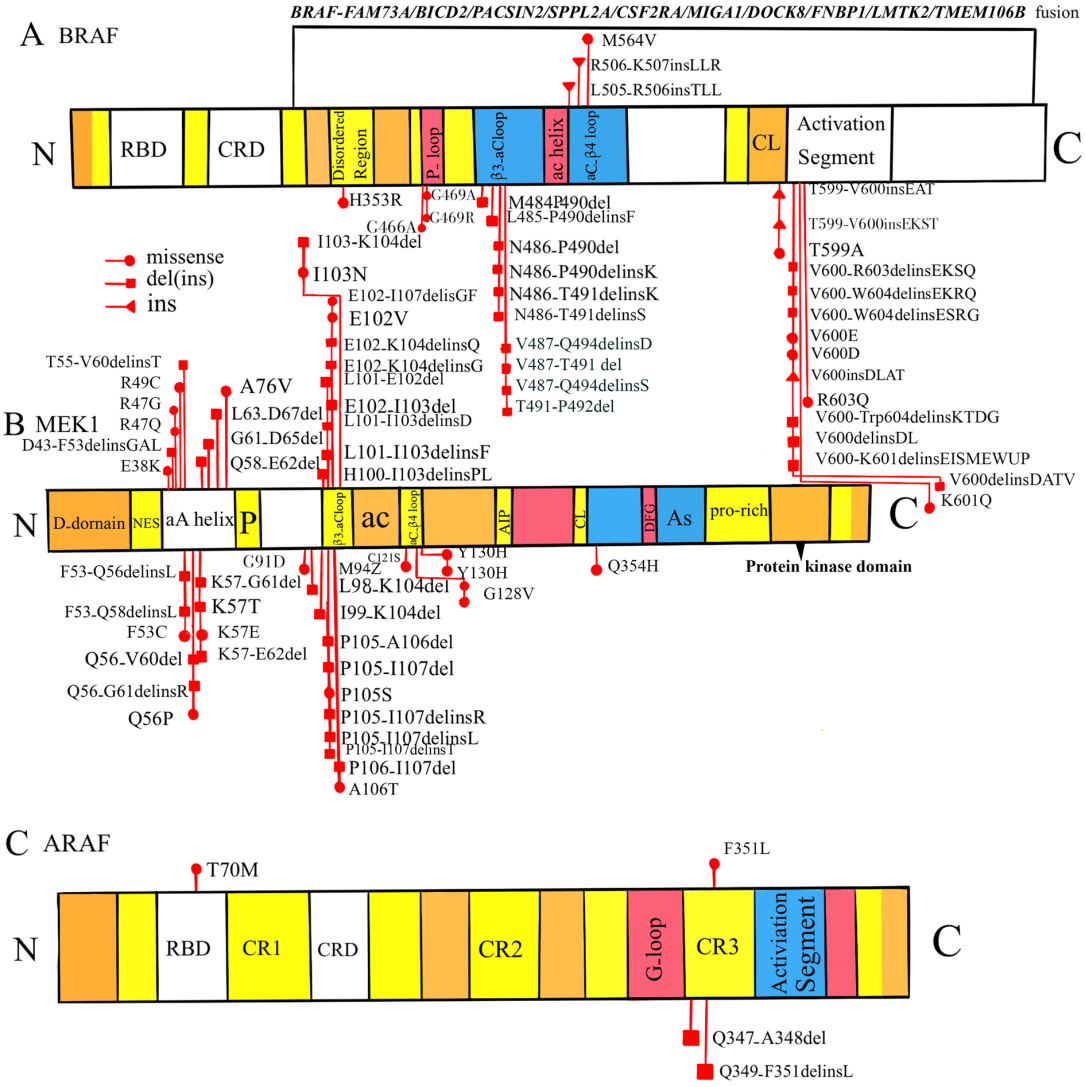
The summary of all alterations of (A) BRAF, (B) MEK1, and (C) ARAF reported in current literature in LCH.

The alterations in BRAF, MEK1, and ARAF in LCH are presented in Figure 2. The types of mutations, listed from top to bottom, included (1) missense mutation, (2) deletion–insertion mutation, (3) deletion mutation, (4) fusion gene, and (5) insertion. Besides the *BRAF* ^*V600E*^ mutation, 31 *BRAF* mutations detected in childhood LCH were recorded, which were predominantly clustered in exon 12 and exon 15, causing modification to the β3‐αC loop and the activation segment of BRAF, respectively. To date, 10 *BRAF* fusion genes have been reported in pediatric LCH. The 49 *MAP2K1* mutations identified in LCH are primarily concentrated in exons 2–3, leading to modifications in the αA helix and β3‐αC loop of MEK1. In comparison, only four *ARAF* mutations have been reported, all occurring in male patients.

**FIGURE 3:**
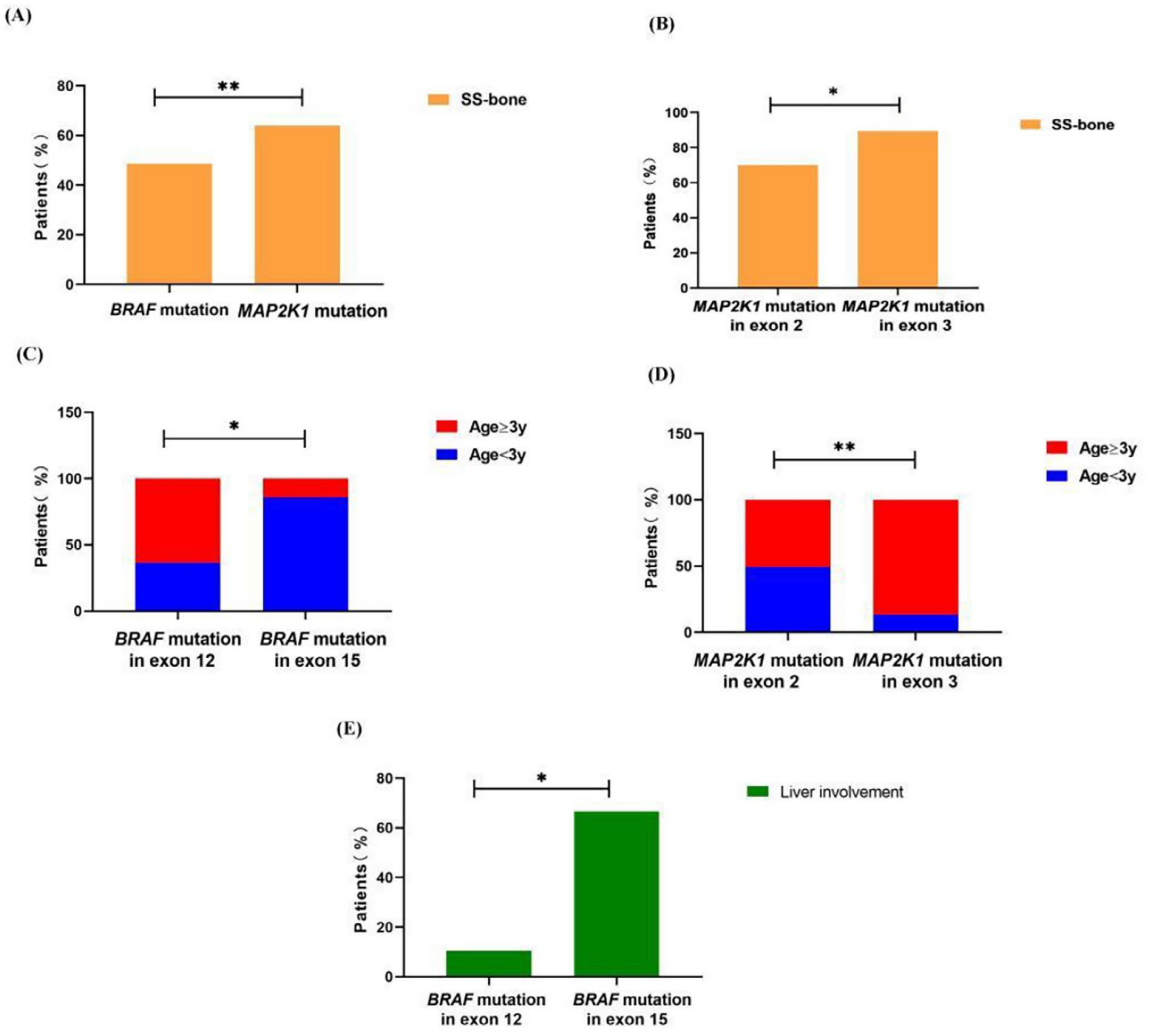
Clinical features of LCH at diagnosis in children with other *BRAF* or *MAP2K1* mutations. (A) Bar charts depicting the percentage of SS‐bone patients with *BRAF* and *MAP2K1* mutations. (B) Bar charts displaying the percentage of SS‐bone patients with *MAP2K1* mutations in exons 2 and 3. (C) Bar charts illustrating the age distribution of patients with *BRAF* mutations in exons 12 and 15. (D) Bar charts depicting the age distribution of patients with *MAP2K1* mutations in exons 2 and 3. (E) Bar charts depicting the percentage of liver involvement with *BRAF* mutation in exons 12 and 15. Statistically significant differences are indicated *P < 0.05, **P < 0.01.

We pooled data from 12 study cohorts and our 31cohorts to obtain a virtual cohort of 303 children with LCH, all genotyped for *MAP2K1* or other *BRAF* alterations. Clinical information for 272 of these patients was retrieved from full‐text articles and/or Supporting Information. The clinical and survival characteristics of patients harboring *MAP2K1* and non‐*BRAF* ^*V600E*^ mutations are summarized in Table 2 and Figure 3. In further analyses, the association between gene mutations and clinical features was explored. This analysis revealed that patients with *MAP2K1* mutations exhibited a higher prevalence of single‐system bone involvement (SS‐bone) compared to those with *BRAF* mutations (*p* = 0.0072). Furthermore, patients with *MAP2K1* mutations in exon 3 had a higher prevalence of SS‐bone than those with *MAP2K1* mutations in exon 2 (*p* = 0.042). In terms of LCH onset, a higher proportion of patients under 3 years of age had *BRAF* mutations in exon 15 and *MAP2K1* mutations in exon 2 compared to those with *BRAF* mutations in exon 12 and *MAP2K1* mutations in exon 3 (*p* = 0.037; *p* = 0.0015). In addition, liver involvement was significantly more frequent in patients with *BRAF* mutations in exon 15 than in those with mutations in exon 12 (*p* = 0.042). However, there were no statistically significant differences in the extent of disease at diagnosis, affected sites, relapse rates, or mortality between patients with *BRAF* and *MAP2K1* mutations.

**TABLE 2:** Clinical characteristics of patients with *MAP2K1* or other‐*BRAF* mutations.

	Other‐*BRAF* mutated			*MAP2K1* mutated			Other‐*BRAF* mutated	*MAP2K1* mutated	*p*
	exon 12	exon 15	*p*	exon 2	exon 3	*p*			
Patients, *n* (%)									
Age < 3 year	20 (36.4)	6 (85.7)	0.037^a^	36 (49.3)	4 (13.3)	0.0015^a^	33 (35.5)	54 (37.2)	0.784^b^
Age ≥ 3 year	35 (63.6)	1 (14.3)		37 (50.7)	26 (86.7)		60 (64.5)	91 (62.8)	
Sex, *n* (%)									
Male	51 (61.4)	4 (57.1)	0.858^a^	43 (64.2)	17 (63.0)	0.899^a^	77 (64.2)	117 (63.9)	0.967^b^
Female	32 (38.6)	3 (42.9)		24 (35.8)	10 (37.0)		43 (35.8)	66 (36.1)	
Disease extent at diagnosis, *n* (%)									
MS	21 (25.0)	4 (36.4)	0.660^a^	15 (22.7)	3 (10.7)	0.254^c^	35 (28.0)	38 (22.9)	0.320^b^
SS	63 (75.0)	7 (63.6)		51 (77.3)	25 (89.3)		90 (72.0)	128 (77.1)	
Detailed subtype, *n* (%)									
Total	62	5		73	28		97	170	
MS‐RO+	8 (14.0)	2 (40.0)	0.157^c^	10 (13.7)	0 (0.0)	0.058^c^	12 (16.2)	14 (10.3)	0.301^a^
MS‐RO−	17 (29.8)	1 (20.0)	> 0.99^c^	12 (16.4)	3 (10.7)	0.550^c^	26 (35.1)	35 (25.7)	0.1585^b^
SS‐bone	32 (56.1)	2 (40.0)	0.922^a^	51 (69.9)	25 (89.3)	0.042^c^	36 (48.6)	87 (64.0)	0.0072^b^
SS‐UFB	26 (45.6)	1 (20.0)	0.526^c^	34 (46.6)	15 (53.6)	0.958^a^	29 (39.2)	76 (55.9)	0.081^b^
SS‐MFB	11 (19.3)	1 (20.0)		14 (19.2)	5 (17.9)		30 (40.5)	45 (33.1)	
Disease site(s) at diagnosis, *n* (%)									
Total	67	3		64	22		100	163	
Bone	66 (98.5)	3 (100)	0.690^a^	63 (98.4)	22 (100.0)	0.964^b^	95 (95.0)	157 (96.3)	0.753^b^
Skin	16 (23.9)	1 (33.3)	0.560^c^	8 (12.5)	0 (0.0)	0.107^c^	18 (18.0)	17 (10.4)	0.093^c^
Liver	7 (10.4)	2 (66.7)	0.042^c^	4 (6.25)	0 (0.0)	0.296^c^	10 (10.0)	7 (4.3)	0.076^c^
Hematopoietic system	2 (3.0)	1 (33.3)	0.125^c^	4 (6.25)	0 (0.0)	0.296^c^	4 (4.0)	6 (3.7)	> 0.99^c^
Spleen	3 (4.5)	1 (33.3)	0.164^c^	3 (4.7)	0 (0.0)	0.549^c^	4 (4.0)	6 (3.7)	> 0.99^c^
Lymph node	11 (16.4)	0 (0.0)	> 0.99^c^	8 (12.5)	5 (22.7)	0.303^c^	13 (13.0)	28 (17.2)	0.298^c^
Lung	13 (19.4)	2 (66.7)	0.114^c^	13 (20.3)	1 (4.5)	0.104^c^	19 (19.0)	24 (14.7)	0.499^c^
Pituitary	3 (4.5)	1 (33.3)	0.164^c^	2 (3.1)	1 (4.5)	> 0.99^c^	9 (9.0)	8 (4.9)	0.205^c^
Relapse, *n*	2	1	0.125^c^	1	1	0.448^c^	6	14	0.637^c^
Death, *n*	1	0	> 0.99^c^	3	0	0.549^c^	1	3	> 0.99^c^

Abbreviations: SS‐MFB, multifocal bone disease; SS‐UFB: unifocal bone disease.

^a^ Continuity correction.

^b^ Pearson chi‐square.

^c^ Fisher's exact test.

## 4 Discussion

This study presents retrospective analyses of the correlation between non‐*BRAF* ^*V600E*^ genotypes and clinical phenotypes in pediatric LCH. Besides *BRAF* ^*V600E*^ mutations, there are several primary molecular alterations in childhood LCH, including mutations in *MAP2K1* exon 2 or 3, *BRAF* exon 12 or 15, and *ARAF*. This study found that *MAP2K1* mutations, particularly those in exon 3, were associated with a higher risk of SS‐bone involvement compared to *BRAF* mutations. Additionally, liver involvement was more frequent in patients with *BRAF* mutations in exon 15 than in those with mutations in exon 12. This suggests that a genotype–phenotype correlation exists among the subtypes of childhood LCH.

Studies have demonstrated that the *BRAF* ^*N486_P490del*^ mutation is the most common in‐frame *BRAF* deletion in adult LCH with pulmonary involvement [33]. However, at our center, only two patients carried the *BRAF* ^*N486_P490del*^ mutation. Both patients were diagnosed with RO+ MS‐LCH and exhibited poor responses to induction chemotherapy. One patient treated with cytarabine, cladribine, or dabrafenib succumbed to tumor progression resulting in liver failure. The other patient was enrolled in a clinical trial investigating the efficacy of a novel MEK inhibitor due to reactivation of disease after induction chemotherapy. A study by Messinger et al., reported that two patients with RO‐MS‐LCH harboring the *BRAF* ^*N486_P490del*^ mutation experienced multiple disease reactivations despite treatment with various chemotherapy regimens. These patients showed good response to trametinib monotherapy, with tolerable toxicity [29]. Similarly, an adult MS‐LCH patient with the *BRAF* ^*N486_P490del*^ mutation showed marked improvement in both soft tissue and central nervous system symptoms after receiving targeted MEK inhibitor therapy [34]. These results indicate that LCH with the *BRAF* ^*N486_P490del*^ mutation often affects multiple organs, has a high likelihood of disease reactivation, and shows a good response to MEK inhibitor treatment.

A follow‐up of 33 patients revealed that the OS (90.9%) and PFS (83.5%) in this cohort of pediatric LCH were comparable to those of all childhood LCH cases treated at our center [11]. Recently, an international clinicogenomic study involving 377 pediatric LCH patients concluded that *BRAF* and *MAP2K1* mutations were not associated with event‐free survival when patients were stratified by disease extent [4]. Similarly, a study on 233 Chinese pediatric LCH patients found that *BRAF* and *MAP2K1* mutations were not independent prognostic factors, regardless of whether the patient received second‐line treatment or targeted therapy [25].

A multicenter clinical study in France involving 366 pediatric LCH found no significant differences in disease characteristics and outcomes between the subgroup with *MAP2K1* mutations (*n* = 44) and that with *BRAF* exon 12 mutation (*n* = 34). However, although patients with non‐*BRAF* ^*V600E*^‐mutated LCH generally exhibited less severe disease, a subset of patients with specific mutations, such as *BRAF* exon 12 small duplications, *BRAF* exon 12 small deletions, *MAP2K1* ^*F53*C^, *MAP2K1* ^*Q56_G61delinsR*^, and *MAP2K1* ^*F53_Q58delinsL*^, presented with life‐threatening conditions. Rencently, another retrospective cohort study from the Netherlands and Canada on pediatric LCH reported 54 patients with *MAP2K1* mutations, 39 with *BRAF* exon 12 mutations, and 13 with rare *BRAF* alterations. The results showed that *MAP2K1* mutations were associated with a higher prevalence of SS‐bone LCH, while *BRAF* exon 12 deletions were correlated with an increased risk of lung involvement [4]. By combining two study cohorts, Kemps et al. established a virtual group comprising 186 children with LCH, genotyped for *MAP2K1* or *BRAF* mutations. Results showed *MAP2K1* mutations predicted bone involvement, whereas *BRAF* exon 12 deletions correlated with lung involvement [32]. By reviewing 303 pediatric LCH cases, we confirmed that *MAP2K1* mutations were associated with higher SS‐bone LCH prevalence. The *MAP2K1* mutations in exons 2 and 3 modify the αA helix and β3‐αC loop of MEK1, while *BRAF* mutations in exons 12 and 15 affect the β3‐αC loop and activation segment of BRAF in childhood LCH. Comparison of LCH characteristics between these mutations showed that patients with *MAP2K1* mutations in exon 3 had a higher prevalence of SS‐bone than those with mutations in exon 2. Patients under 3 years old tended to have more *MAP2K1* mutations in exon 2 than in exon 3, and similarly, they had more *BRAF* mutations in exon 15 than in exon 12. Furthermore, liver involvement was more frequent in patients with *BRAF* mutations in exon 15 compared to those with exon 12.

In conclusion, *MAP2K1* mutations, especially in exon 3, were more frequent in SS‐bone than *BRAF* mutations. Liver involvement was higher in patients with *BRAF* mutations in exon 15 compared to exon 12. However, it is essential to acknowledge that our analysis is limited to the clinical features documented in the retrieved studies, which exhibit incomplete information and heterogeneity in molecular testing, as well as the results derived from those samples. Thus, we could not ascertain the current survival status of these patients. Therefore, to better elucidate the clinical impact of recurrent mutations beyond *BRAF* ^*V600E*^ in childhood LCH and validate the present findings, larger cohorts of patients should be enrolled through international collaborations.

## Author Contributions

X.T. wrote the manuscript. X.T., Z.W., and J.‐j.S. contributed to patient follow‐up. X.T., J.G., and X.G. contributed to the data collection. X.T., Z.W., and J.‐j.S. contributed to data analysis. J.G. and X.G. contributed to study conception and design. J.G. and X.G. revised the manuscript. All authors revised the paper and approved the submitted version.

## Ethics Statement

This study was approved by the Ethics Committee of the West China Second University Hospital of Sichuan University (No. 164).

## Consent

The authors have nothing to report.

## Conflicts of Interest

The authors declare no conflicts of interest.

## Supporting information


Appendix S1.

## Data Availability

The datasets generated and/or analyzed during the current study are available. in the Web of Science and PubMed repositories (webofScience.com, pubmed.ncbi.nlm.nih.gov), and the datasets used during the current study are available from the corresponding author on reasonable request.
