# Supplementary material for: Beyond BRAFV600E : Investigating the Clinical and Genetic Spectrum of Langerhans Cell Histiocytosis in Children
Source: Cancer Med. 2024 Dec 23;13(24):e70532. doi: 10.1002/cam4.70532 (PMC11664316; doi:10.1002/cam4.70532)
Supplement: Supplementary file 1 — Appendix S1. [file CAM4-13-e70532-s001.docx]

| **Supplemental Table 1** Genes and fusion genes in the Panel of our study | | | | | |
| --- | --- | --- | --- | --- | --- |
| AKT1 | ALK | ARAF | ASXL1 | BRAF | CBL |
| CCND1 | CD36 | CDKN2A | CSF1R | CSF3R | DDR2 |
| EGFR | ERBB2 | ERBB3 | FGFR1 | FGFR2 | HRAS |
| IDH2 | KIT | KRAS | MAP2K1 | MAP2K2 | MAP3K1 |
| MET | MITF | NOTCH1 | NRAS | NTRK1 | PDGFRA |
| PBX1 | PIK3CA | PIK3CD | PTEN | RAF1 | RET |
| ROS1 | SMAD6 | SMO | SOX2 | TP53 | TSC1 |
| U2AF1 | PLEKHA6-NTRK3 | PACSIN2-BRAF | FAM73A-BRAF | BICD2-BRAF | |

| **Supplemental Table 2** The Clinical characteristics and experimental results of 33 LCH patients | | | | | | | | |
| --- | --- | --- | --- | --- | --- | --- | --- | --- |
| **ID.**  **no** | **Age/sex** | **Disease site(s)** | **Risk**  **category** | **Clinical**  **stage** | **Type of aberration** | | | **Survival status** |
|  |  |  |  |  | **Gene** | **RNA level** | **Protein level** |  |
| 1 | 5y/F | Bone | Low | SS | *BRAF* | c.1690A>G | p.M564V | Survival |
| 2 | 10y/F | Bone | Low | SS | *BRAF* | c.1455_1469del | p.L485_490delinsF | Survival |
| 3 | 14y/M | Bone, thymus | Low | MS | *BRAF;*  *MAP3K1* | c.1455_1469del;  c.2012C>T | p.L485_490delinsF;  p.A671V | Survival |
| 4 | 11y/M | Bone, muscle | Low | MS | *BRAF* | c.1455_1469del | p.L485_490delinsF | Survival |
| 5 | 1y/F | Bone | Low | SS | *BRAF* | c.1455_1469del | p.L485_490delinsF | Survival |
| 6 | 9y/M | Nasopharynx, submandibular gland, lymph nodes, thymus, stomach, liver | High | MS | *BRAF* | c.1457_1471del | p.N486_P490del | Survival |
| 7 | 2y/M | Liver, spleen, lung, ears, skin, nails, toenails, lymph nodes,right diaphragmatic angle | High | MS | *BRAF* | c.1457_1471del | p.N486_P490del | Death |
| 8 | 1y/M | Bone | Low | SS | *BRAF* | c.1458_1472del | p.N486_T491delinsK | Survival |
| 9 | 5y/M | Bone | Low | SS | *BRAF* | c.1458_1472del | p.N486_T491delinsK | Survival |
| 10 | 6y/M | Bone | Low | SS | *BRAF* | c.1458_1472del | p.N486_T491delinsK | Survival |
| 11 | 1y/M | Bone, lymph nodes | Low | MS | *BRAF* | c.1511_1517+2dup | p.R506_K507insLLR | Survival |
| 12 | 6m/M | Nasal cavity, left upper alveolar, bones, lung, liver | High | MS | *BRAF* | c.1799_1800delinsAT | p.V600D | Survival |
| 13 | 1y/M | Bone, skin | Low | MS | *KRAS* | c.35G>C | p.G12A | Survival |
| 14 | 8y/M | Bone | Low | SS | *MAP2K1* | c.167A>C | p.Q56P | Survival |
| 15 | 14y/M | Pituitary gland, skin, ear | Low | MS | *MAP2K1* | c.167A>C | p.Q56P | Survival |
| 16 | 8m/M | Bone, skin | Low | MS | *MAP2K1* | c.159_173del | p.F53_Q58delinsL | Death |
| 17 | 1y/M | Bone, liver, spleen, lung, lymph nodes, hematopoietic system | High | MS | *MAP2K1* | c.159_173del | p.F53_Q58delinsL | Survival |
| 18 | 6y/F | Bone | Low | SS | *MAP2K1* | c.159_173del | p.F53_Q58delinsL | Survival |
| 19 | 5m/M | Bone, lung, hematopoietic system | High | MS | *MAP2K1* | c.159_173del | p.F53_Q58delinsL | Survival |
| 20 | 10y/F | Bone | Low | SS | *MAP2K1* | c.171_185del | p.Q58_E62del | Survival |
| 21 | 5y/M | Bone | Low | SS | *MAP2K1* | c.171_185del | p.Q58_E62del | Survival |
| 22 | 11y/F | Bone | Low | SS | *MAP2K1* | c.171_185del | p.Q58_E62del | Survival |
| 23 | 11 y/F | Bone | Low | SS | *MAP2K1* | c.171_185del | p.Q58_E62del | Survival |
| 24 | 7y/M | Bone | Low | SS | *MAP2K1* | c.173_187del | p.Q58_E62del | Survival |
| 25 | 1y/F | Bone, lung | Low | MS | *MAP2K1* | c.173_187del | p.Q58_E62del | Survival |
| 26 | 2y/M | Bone | Low | SS | *MAP2K1* | c.173_187del | p.Q58_E62del | Survival |
| 27 | 12y/M | Bone | Low | SS | *MAP2K1* | c.173_187del | p.Q58_E62del | Survival |
| 28 | 6y/M | Bone | Low | SS | *MAP2K1* | c.165_179del | p.Q56_V60del | Survival |
| 29 | 2y/F | Bone, lung, lymph nodes | Low | MS | *MAP2K1* | c.173_187del | p.Q58_E62del | Survival |
| 30 | 11y/M | Bone | Low | SS | *MAP2K1* | c.303_308del | p.E102_I103del | Survival |
| 31 | 6y/M | Bone | Low | SS | *MAP2K1* | c.304_309del | p.E102_I103del | Survival |
| 32 | 2y/F | Lymph nodes, thyroid gland | Low | MS | *MAP2K1* | c.305_311delinsG | p.E102_K104delinsG | Survival |
| 33 | 2y/M | Diaphragm, thyroid gland, lymph nodes, esophagus, lung | Low | MS | *ARAF* | c.1046_1051del | p.Q349_F351delinsL | Survival |

| 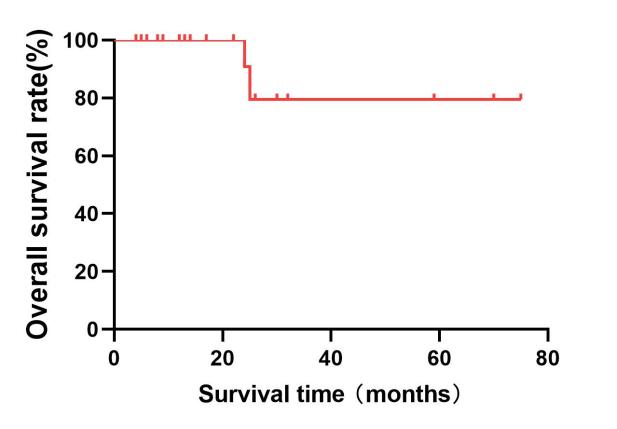 | 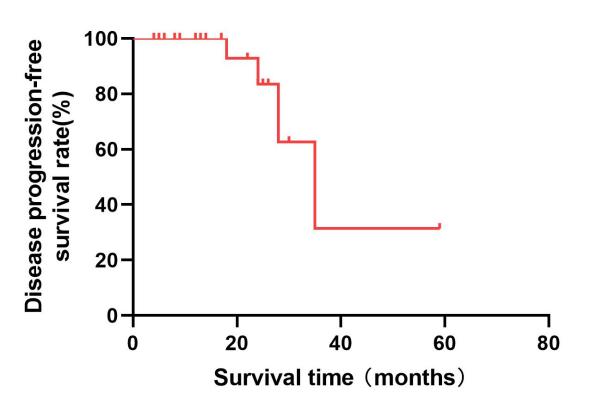 |
| --- | --- |
| **Supplemental Fig. 1** Kaplan-Meier’s curves for OS and PFS of our 33 patients | |
